# LACCASE14 is required for the deposition of guaiacyl lignin and affects cell wall digestibility in poplar

**DOI:** 10.1186/s13068-020-01843-4

**Published:** 2020-12-03

**Authors:** Shifei Qin, Chunfen Fan, Xiaohong Li, Yi Li, Jian Hu, Chaofeng Li, Keming Luo

**Affiliations:** 1 Chongqing Key Laboratory of Plant Resource Conservation and Germplasm Innovation, School of Life Sciences, Southwest University, No. 2, Tiansheng Road, Beibei, 400716 Chongqing China; 2 Key Laboratory of Eco-Environments of Three Gorges Reservoir Region, Ministry of Education, School of Life Sciences, Southwest University, Chongqing, 400715 China

**Keywords:** *Populus*, Dwarf, Laccase, Lignin, Monolignol, Guaiacyl unit, Saccharification

## Abstract

**Background:**

The recalcitrance of lignocellulosic biomass provided technical and economic challenges in the current biomass conversion processes. Lignin is considered as a crucial recalcitrance component in biomass utilization. An in-depth understanding of lignin biosynthesis can provide clues to overcoming the recalcitrance. Laccases are believed to play a role in the oxidation of lignin monomers, leading to the formation of higher-order lignin. In plants, functions of only a few laccases have been evaluated, so little is known about the effect of laccases on cell wall structure and biomass saccharification.

**Results:**

In this study, we screened a gain-of-function mutant with a significant increase in lignin content from *Arabidopsis* mutant lines overexpressing a full-length poplar cDNA library. Further analysis confirmed that a Chinese white poplar (*Populus tomentosa*) laccase gene *PtoLAC14* was inserted into the mutant, and *PtoLAC14* could functionally complement the *Arabidopsis lac4* mutant. Overexpression of *PtoLAC14* promoted the lignification of poplar and reduced the proportion of syringyl/guaiacyl. In contrast, the CRISPR/Cas9-generated mutation of *PtLAC14* results in increased the syringyl/guaiacyl ratios, which led to integrated enhancement on biomass enzymatic saccharification. Notably, the recombinant PtoLAC14 protein showed higher oxidized efficiency to coniferyl alcohol (precursor of guaiacyl unit) in vitro.

**Conclusions:**

This study shows that PtoLAC14 plays an important role in the oxidation of guaiacyl deposition on cell wall. The reduced recalcitrance of the *PtoLAC14*-KO lines suggests that PtoLAC14 is an elite target for cell wall engineering, and genetic manipulation of this gene will facilitate the utilization of lignocellulose.

## Background

The plant cell wall represents an enormous renewable biomass resource for biochemical, biofuels and materials production on the earth [1, 2]. Plant cell wall is composed mainly of cellulose, hemicelluloses and lignin, as well as minor pectic polysaccharides and wall proteins [3]. Cellulose and hemicellulose can be converted to bioethanol, biobutanol, and other products. For effective utilization of these carbohydrates, it is necessary to overcome the recalcitrance of cell wall. Lignocellulose recalcitrance is principally determined by cell wall composition, wall polymer feature (such as cellulose crystalline index, degree of polymerization, monomer proportions of lignin), and network among cell wall polymer [4, 5]. Several studies indicate that lignin quantity and quality showed significant impact on the utilization and degradability of biomass [6–8]. During the biological conversion processes, lignin does not only act as a physical barrier to prevent enzyme access to carbohydrates, but also reduces the activity of the enzyme by non-productive binding [9–11]. Lignin content showed negative correlations with cellulose accessibility and sugar release from woody plants and other bioresources [7, 12–14]. Moreover, the lignin composition also impacts usability of lignocellulosic biomass, as syringyl lignin is relatively easier to remove from cell walls than guaiacyl lignin in pretreatment processes [15–18]. Therefore, an in-depth understanding of lignin biosynthesis is required for improving the utility of lignocellulosic biomass.

Lignin is a complex polyphenolic heteromorphic polymer created through the polymerization of the monolignols coniferyl alcohol, p-coumaryl alcohol and sinapyl alcohol that form the guaiacyl (G), hydroxy-coumaryl (H) and syringyl (S) subunits, respectively [19]. The proportion of those three lignin monomer units varies greatly between different plant species. Lignin in both monocots and dicots primarily contains G and S units. Dicot lignin contains undetectable level of H units, whereas monocots lignin has larger amounts of H units [19]. Lignin biosynthesis consists of three major steps, including monolignol biosynthesis in the cytosol, transport of monolignols to the cell wall matrix, and polymerization into the heterogeneous, cross-linked lignin polymer in the cell wall [19, 20]. Each monolignol loses one H atom to produce the free radical forms, and then these free radicals are linked together to form the complex lignin structure [21]. Both laccases and peroxidases are proposed to be involved in monolignol polymerization.

Laccases are copper-containing glycoproteins, which are known to function in oxidation reactions involving various inorganic and organic substrates including phenolics and aromatic amines in plants [22]. Several laccases are involved in lignin biosynthesis. For instance, overexpression of cotton *GaLACCASE 1* (*LAC1*) in *Populus*, led to increased total lignin content [23]. In *Arabidopsis*, the *lac4* or *lac17* single mutants resulted in only slightly reduced lignin content, whereas the *lac4-2/lac17* double mutant displays up to 40% less lignin in the stem [24]. In poplar, *PtLAC3* is essential for cell wall structure and integrity in xylem fibers [25], in addition, knockdown of *PtLAC2* confers altered cell wall chemistry and increased sugar release [26]. Plant laccases belong to a complex protein family, although several attempts have been made to identify the physiological role of different laccase isoforms [25–28], it has remained difficult to assign specific functions to an individual laccase, due to redundancy and wide substrate specificities.

In this study, we identify a poplar PtoLAC14, which is involved in lignin deposition. Overexpression of *PtoLAC14* in *Arabidopsis* resulted in a typical dwarf phenotype, along with increased lignin content. Meanwhile, *PtoLAC14* was successfully used to complement the *Arabidopsis lac4* mutant. We further characterized the role of *PtoLAC14* in secondary cell wall biosynthesis and biomass enzymatic saccharification in poplar. The polymerization activity on monolignols of PtoLAC14 is also evaluated in vitro. Our data show that PtoLAC14 is essential for lignin polymerization by selectively promoting guaiacyl (G) subunit deposition, and has a certain impact on the biomass saccharification in poplar.

## Results

### Morphological characterization of *df1* mutant

**Fig. 1:**
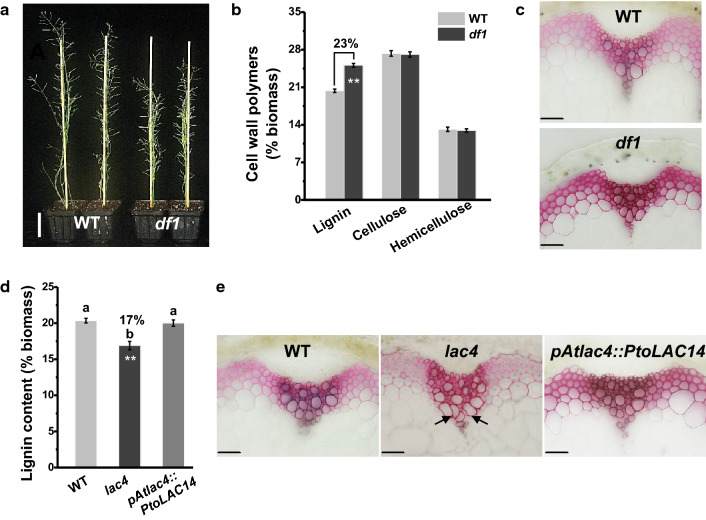
Phenotype of laccase mutants. **a** Growth phenotype of WT and *df1* mutant. **b** Cell wall composition (% biomass) of WT and *df1* mutant. **c** Phloroglucinol–HCl staining of stem cross sections from WT and *df1* mutant. **d** Cell wall composition (% biomass) of WT, *lac4* mutant, and *pAtLAC4::PtoLAC14* transgenic line. **e** Phloroglucinol–HCl staining of stem cross sections from WT, *lac14* mutant, and *pAtLAC4::PtoLAC14* transgenic line. The black arrows show collapsed xylem. Scale bar is 5 cm in a, 100 μm in **c** and **e**. Data represent mean ± SD of three technical replicates. Statistical analyses were performed using Student’s *t* test as **P < 0.01

To identify genes involved in cell wall formation, we constructed a full-length cDNA library from various tissues in *P. tomentosa* and performed full-length cDNA overexpressor (FOX) gene hunting in *Arabidopsis* [29, 30]. A gain-of-function mutant line was generated and named *df1* (*dwarf1*), which showed dwarf and thin inflorescence stems phenotype (Fig. 1a). As plant cell walls represent major components of stems, we measured cell wall composition of *df1*. By comparison, the *df1* mutant contained significantly more lignin level than that of the wild-type (WT) at *P* < 0.01 level, but with a similar cellulose and hemicelluloses content (Fig. 1b). Moreover, the cross sections of stems were stained with the phloroglucinol–HCl reagent. The stronger staining signals of sclerenchyma tissues further revealed an increased lignin accumulation in the *df1* mutant (Fig. 1c), suggesting that the *df1* gene promoted lignin biosynthesis.

### Identification of the *df1* gene

To clone the inserted full-length cDNA fragment, specific primers for the FOX hunting system vector were designed (Additional file 1: Table S1). After amplification and sequencing, a laccase gene (designated *PtoLAC14*) was identified [31]. PtoLAC14 contains four conserved copper-binding sites and a predicted N-terminal signal peptide for secretion (Additional file 1: Fig. S1). Multiple sequence alignment revealed that PtoLAC14 shared high identity with characterized laccase proteins, including AtLAC4 and AtLAC17 from *Arabidopsis* [24], PtLAC2 and PtLAC3 from poplar [25, 26], BdLAC5 from *Brachypodium distachyon* [32, 33], and SofLAC from sugarcane [34] (Additional file 1: Fig. S1, Table S2). To verify the function of *PtoLAC14*, several independent homozygous lines expressing *PtoLAC14* driven by *AtLAC4* promoter (*pAtLAC4::PtoLAC14*) in the *Atlac4* mutant background were generated, and the expression of *PtoLAC14* was confirmed by RT-PCR (Additional file 1: Table S1, Fig. S2). As previously reported, the *Atlac4* mutant has lower lignin content when compared to wild-type plants [24]. The total lignin content of wild-type *Col-0* plants was 20.4%, but decreased to 16.9% in the *Atlac4* mutant (Fig. 1b). In comparison, in the complementary lines expressing *pAtLAC4::PtoLAC14*, total lignin content was restored to the WT levels (Fig. 1d). Furthermore, using HCl–phloroglucinol staining of stem cross sections, complete reversal of the irregular xylem phenotype in *Atlac4* mutant was apparent in the lines complemented with *pAtLAC4::PtoLAC14* (Fig. 1e). Together, the results suggest that *PtoLAC14* was involved in the regulation of lignin deposition.

### Retarded plant growth and stem development in *PtoLAC14*-OE lines

In poplar, *PtoLAC14* was highly expressed in the typically lignified tissues, such as xylem in stem (Additional file 1: Fig. S3). To investigate the function of *PtoLAC14* in growth and development of poplar, we generated the *PtoLAC14*-overexpressed (OE) plants. *PtoLAC14* transcript levels were determined by qPCR, and three independent lines displaying high expression levels (OE-L1, L2, and L5) were selected for comparative analyses (Additional file 1: Fig. S4 a-c). Consistent with the expression levels of *PtoLAC14*, the *PtoLAC14*-OE lines displayed a larger increase in laccase activity (Additional file 1: Fig. S4 d).

**Fig. 2:**
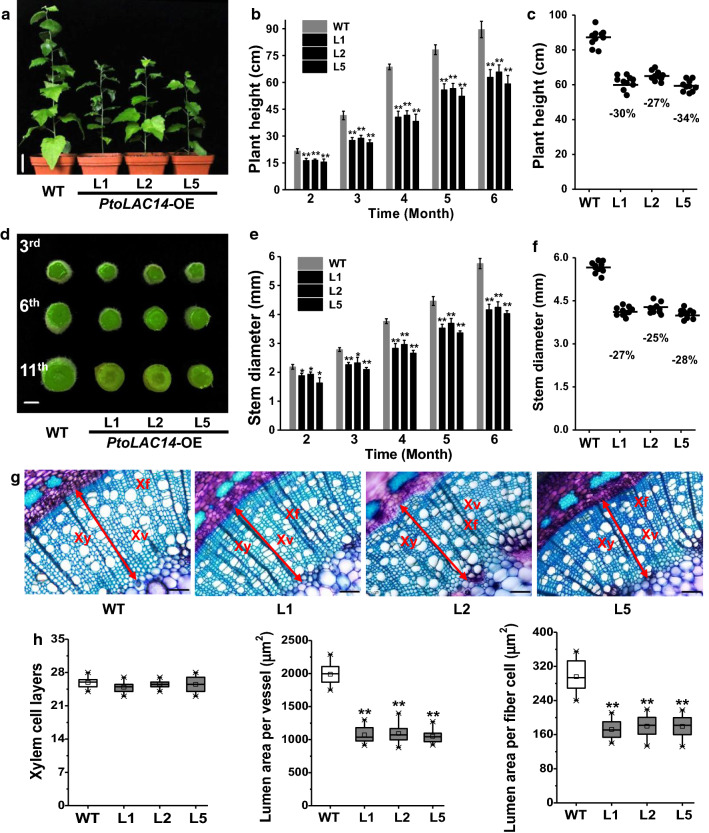
Measurement of plant growth in *PtoLAC14*-OE transgenic poplar plant. **a** Images of 6-month-old *PtoLAC14*-OE transgenic lines and wild type (WT); scale bar is 10 cm. **b** Plant height in the *PtoLAC14*-OE transgenic lines and WT during time course of 6 months. **c** Plant height in *PtoLAC14*-OE transgenic lines and WT of 6-month-old. **d** Observation of stem diameter in *PtoLAC14*-OE transgenic lines and WT of 6-month-old. **e** Stem diameter in the transgenic lines and WT during time course of 6 months. **f** Stem diameter in *PtoLAC14*-OE transgenic lines and WT of 6-month-old. **g** Toluidine blue staining of the 6th internode stems of 6-month-old *PtoLAC14*-OE lines and WT (Xy: xylem, Xf: xylem fiber cells, Xv: xylem vessel. Scale bar is 100 μm). **h** Numbers of xylem cell layers (cell files) and lumen area of individual xylem vessel cell and fiber cell. Data represent mean ± SD (*n* = 10 in b, c, e, f, and *n* = 30 in h). Student’s *t*-test was performed between the transgenic lines and WT as **P < 0.01

During the 6-month period of growth, the *PtoLAC14*-OE lines maintained much retarded growth compared to the WT, including decreased plant height and stem diameter (Fig. 2a–f). With respect to the changed stem growth in the *PtoLAC14*-OE transgenic plants as described above, this study observed stem morphology. Compared to WT, the *PtoLAC14*-OE transgenic lines exhibited significantly decreased stem diameter, as well as smaller vessel and fiber cells at 45%–47%, 40%–42%, respectively, whereas the xylem layer was not changed (Fig. 2 g–h). Hence, the results suggest that overexpression of *PtoLAC14* leads to a reduction in cell sizes, yielding relatively decreased plant heights and stem diameter in transgenic lines.

### Modified cell wall disposition and altered monomer lignin content in *PtoLAC14*-OE lines

**Fig. 3:**
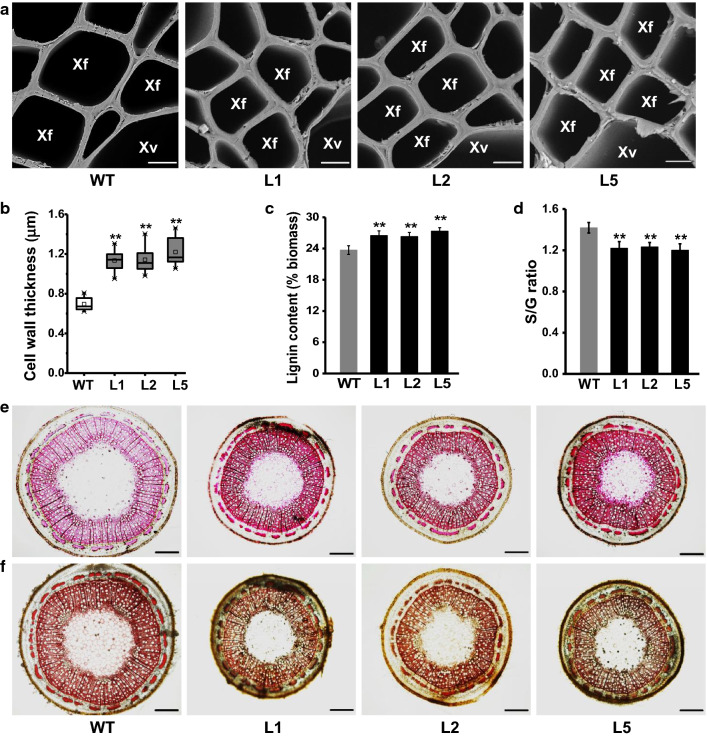
Observations of plant cell walls in *PtoLAC14*-OE transgenic poplar plant. **a** Scanning electron microscopy (SEM) images (Xy: xylem, Xf: xylem fiber cells, Xv: xylem vessel. Scale bar is 10 μm). **b** Cell wall thickness of SEM observation. **c** Lignin content (% biomass). **d **The monomer lignin S/G ratio. **e** Phloroglucinol–HCl staining of stem cross sections from WT and *PtoLAC14*-OE lines. **f** Mäule staining for lignin monomer composition of stem cross sections from WT and *PtoLAC14*-OE lines. Scale bars are 100 μm in **e** and **f**. All data as means ± SD (*n* = 30 in b, *n* = 3 in **c** and **d**). Student’s *t*-test was performed between the transgenic line and WT as **P < 0.01

We then observed thickened cell walls in xylem tissues of *PtoLAC14*-OE transgenic lines (Fig. 3a), and measured significantly increased cell wall width by 63%-70% relative to WT plants (Fig. 3b), indicating that overexpression of *PtoLAC14* leads to a remarkably increased secondary cell wall thickness in transgenic plants.

As plant cell walls represent major components of biomass, cell wall composition of the transgenic plants was measured. By comparison, the *PtoLAC14*-OE transgenic lines contained significantly more lignin levels than those of the WT by 11%-15% at *P* < 0.01 levels (Fig. 3c), but with a similar cellulose and hemicelluloses content (Additional file 1: Table S3). In terms of the increased lignin levels, the three transgenic lines exhibited significantly increased G and H-monomer content, as compared with WT (Additional file 1: Table S4). In particular, the S/G ratio was 13% to 15% lower in *PtoLAC14*-OE transgenic lines, compared with WT (Fig. 3d), suggesting that the *PtoLAC14* predominantly affected the total lignin and lignin monomer content in the *PtoLAC14*-OE plants. Accordingly, we observed increased histochemical staining in xylem tissues of the *PtoLAC14*-OE lines (Fig. 3e), in supporting for the increased lignin levels. Staining with the Mӓule reagent, which is a histochemical stain for qualitative evaluation of lignin monomer content in plants [35], produced a red coloration in the WT control, but a brown staining in *PtoLAC14*-OE stems (Fig. 3f), consistent with previous biochemical analysis of increased G levels in the *PtoLAC14*-OE lines. Therefore, these data demonstrated that the *PtoLAC14*-OE plants had significantly increased lignin levels, and changed S- and G-monomer relative to WT plants.

### Increased S/G ratio in *PtoLAC14*-KO lines

In order to further examine the role of *PtoLAC14* in lignin biosynthesis, we generated targeted mutants of *PtoLAC14* via the CRISPR/Cas9 system (Additional file 1: Fig. S5a), according to our previous report [36]. The PCR-amplified products were detected by sequencing analysis of randomly selected clones from individual transgenic plants (Additional file 1: Fig. S5b-e). The results revealed that there were deletions at the desired target sites caused by the CRISPR/Cas9 system, introducing indels into the *PtoLAC14* gene. Among them, sequence rearrangements were detected in transgenic lines L1 and L3, leading to translational frameshift of *PtoLAC14* (Additional file 1: Fig. S5e). Moreover, in accordance with the simultaneous disruption of *PtoLAC14*, the *PtoLAC14* mutant lines displayed a decrease in laccase activity (Additional file 1: Fig. S5f). These results indicated that heritable mutations were generated in the *PtoLAC14*-KO lines.

**Fig. 4:**
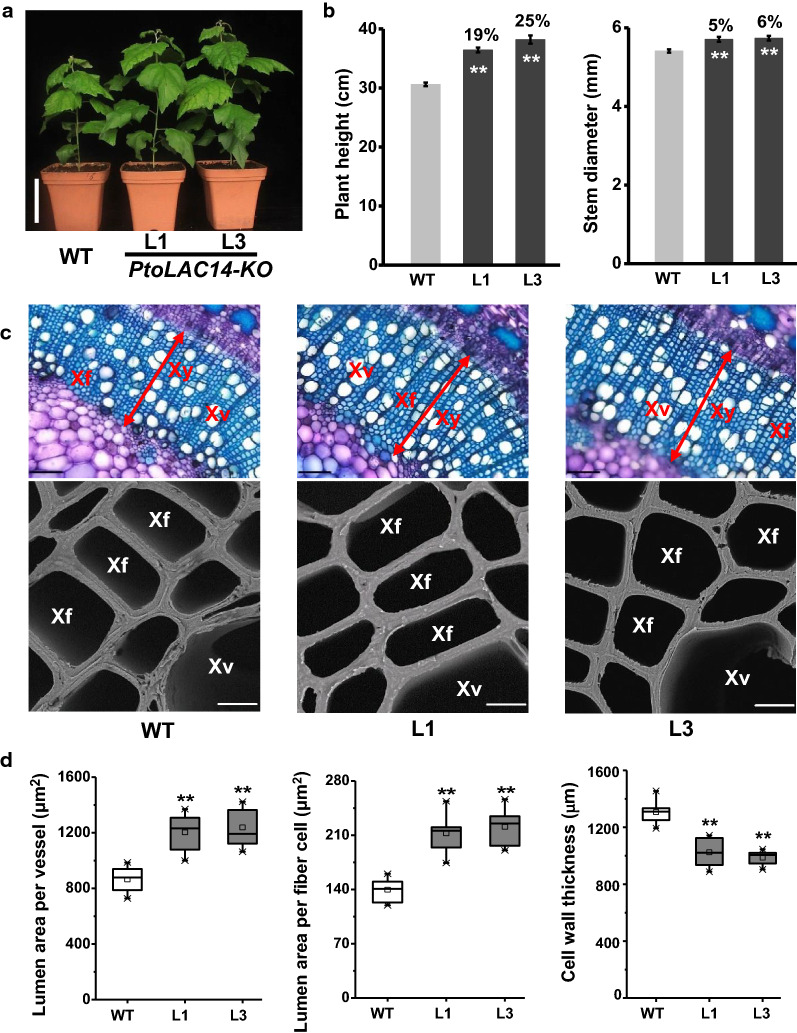
Phenotype of *PtoLAC14*-KO transgenic poplar lines. **a** Images of 3-month-old *PtoLAC14*-KO lines and WT; scale bar is 10 cm. **b** Plant height and stem diameter in the *PtoLAC14*-KO and WT of 3-month-old. **c** Toluidine blue staining and scanning electron microscopy (SEM) images (Xy: xylem, Xf: xylem fiber cells, Xv: xylem vessel. Scale bars are 100 μm and 10 μm, respectively). **d** Lumen area of individual xylem vessel cell, fiber cell, and cell wall thickness. Data represent mean ± SD (*n* = 3 in b and *n* = 30 in **d**). Student’s *t*-test was performed between the transgenic lines and WT as **P < 0.01

After three months of growth, we observed small changes in plant growth and development in all *PtoLAC14*-KO lines. Compared with WT, the plant height and stem diameter were slightly increased in *PtoLAC14*-KO transgenic plants (Fig. 4a, b). Accordingly, the *PtoLAC14*-KO transgenic lines exhibited much expanded xylem area with larger vessel and fiber cells, while the xylem layer was not changed (Fig. 4c, d). Reduced cell wall thickness was also observed in *PtoLAC14*-KO plants (Fig. 4c, d). Specifically, lower concentrations of G units were detected, resulting in a significant an increased S/G ratio in *PtoLAC14*-KO lines, compared to WT plants (Additional file 1: Fig. S6, Table S4).

### PtoLAC14 oxidizes monolignols in vitro

**Fig. 5:**
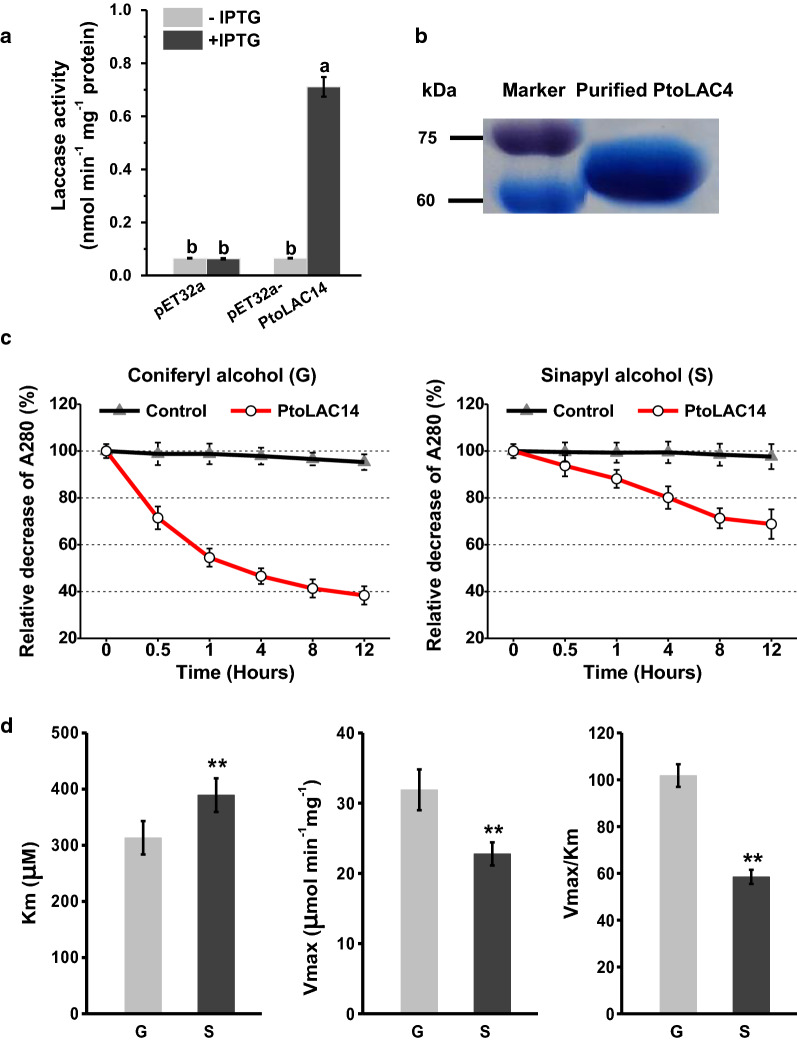
Enzymatic oxidation of monolignols by recombinant PtoLAC14 protein. **a** Quantification of PtoLAC14 activity in E. coli, with ABTS as the substrate. **b** The purified recombinant PtoLAC14 protein analyzed by SDS-PAGE. **c** The activity of purified recombinant PtoLAC14 protein, with coniferyl alcohol and sinapyl alcohol as the substrate. **d** Kinetic properties of PtoLAC14 in vitro. Km and Vmax were determined by linear regression of v against v/s (Eadie–Hofstee plot) with at least five data points. The data are mean values from three independent measurements

To determine the enzymatic activity of PtoLAC14, we produced recombinant protein in *E. coli*. Kinetically, the PtoLAC14 showed laccase activity (Fig. 5a). To understand the catalytic properties of PtoLAC14, the recombinant PtoLAC14 was purified, and the oxidation of different monolignols were measured by determining the decrease of different substrates (Fig. 5b, d). While recombinant PtoLAC14 enzyme was able to oxidize all supplemented monolignols, coniferyl alcohol (precursor of guaiacyl lignin) was oxidized with the higher efficiency than sinapyl alcohol (precursor of syringyl lignin) (Fig. 5b, c). As mentioned above, we showed that overexpression of *PtoLAC14* resulted in significantly higher production of lignin guaiacyl (G) unit in transgenic plants (Fig. 3, Additional file 1: Table S4), whereas inhibition of PtoLAC14 activity reduced the accumulation of G units (Additional file 1: Fig. S6, Table S4). These results together suggest that the PtoLAC14 is involved in the regulation of lignin monomer composition, and preferentially uses G units during polymerization.

### Enhanced cell wall digestibility and sugar yield in *PtoLAC14*-KO lines

**Fig. 6:**
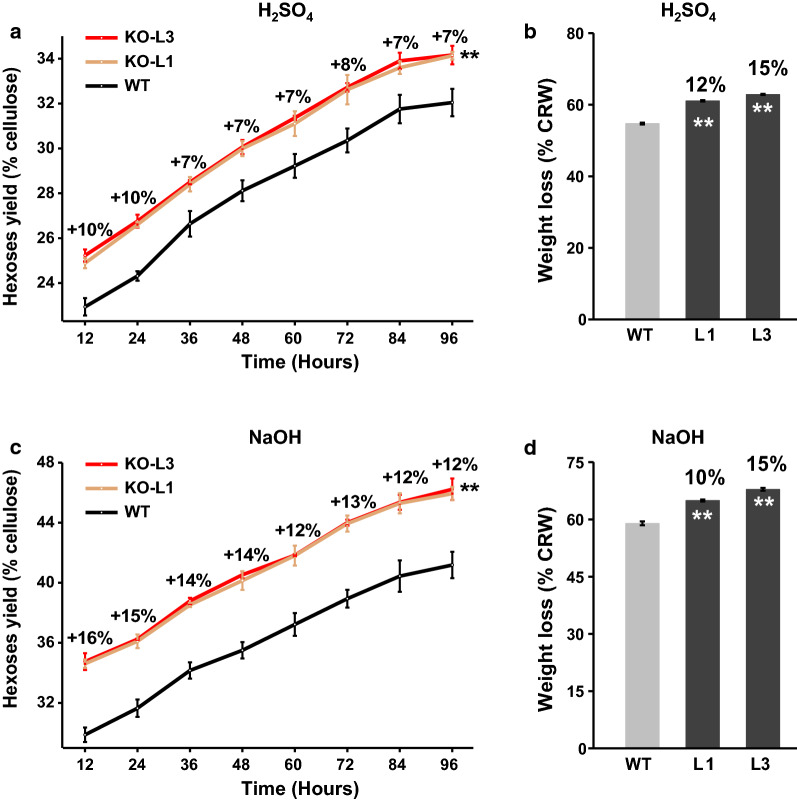
Analyses of biomass enzymatic saccharification in the *PtoLAC14*-KO lines and WT. **a** Hexose yields released from enzymatic hydrolysis after the pretreatment with 4% H_2_SO_4_, **b** 4% NaOH pretreatments. **c** Digestive weight loss after enzymatic hydrolysis and the pretreatment with 4% H_2_SO_4_, **d** 4% NaOH pretreatments. CWR, cell wall residues. Data represent mean ± SD of three technical replicates. Student’s *t*-test between transgenic line and WT as **P < 0.01

Lignin has been considered as one of the crucial factors negatively affecting biomass saccharification and bioethanol production [37]. As lignin content and composition were altered, we next measured cell wall digestibility in the transgenic plants by calculating hexose yields (% biomass) released from enzymatic hydrolysis under two mild pretreatment condition. As shown in Fig. 6, the *PtoLAC14*-KO plants, which contained high S/G ratios, exhibited an increase of 7%–10% in hexoses release from 12 h to 96 h of enzymatic hydrolysis upon 4% H2SO4 pretreatment, and also yielded more hexoses with an increase of 12%–16% upon 4% NaOH pretreatment, compared with WT plants (Fig. 6a, b). In addition, total hydrolysis obtained from cell walls was also quantified based on the loss of dry weight, and all of these *PtoLAC14*-KO transgenic lines displayed 10%–15% increase in the loss of dry weight, comparison with WT plants (Fig. 6c, d). Our data suggest that knock out *PtoLAC14* could largely enhance biomass digestibility and saccharification.

## Discussion

In plants, laccases have been identified as key enzymes in lignin biosynthesis based on their capability to oxidize lignin precursors. For example, lignin deposition in roots is almost completely abolished in the *Arabidopsis lac4/lac11/lac17* triple mutant [38], while overexpression of a cotton laccase (GaLACCASE 1) in *Populus* leads to increased lignin content at 2.1%–19.6% [23]. In this study, a laccase isoform closely related to *AtLAC4* was cloned from poplar and named *PtoLAC14*. The rescue of the collapsed xylem vessels phenotypes, together with the restored lignin content in the complemented *Atlac4* mutant (Fig. 1), confirm that PtoLAC14 can functionally replace the combined activities of the *Arabidopsis* laccase AtLAC4. Beyond this, *PtoLAC14* transcripts were expressed primarily in lignifying tissues (Additional file 1: Fig. S3), and the overexpression of *PtoLAC14* promoted the lignification in poplar (Fig. 3c). These results strongly support the key role of *PtoLAC14* on lignin deposition during wood formation.

As the total lignin levels in transgenic lines were altered, the composition of lignin was also examined. Compared to WT, the *PtoLAC14*-OE lines showed significantly increased G lignin, with reduced S/G ratios (Fig. 3d, f, Additional file 1: Table S4). Inversely, *PtoLAC14*-KO lines showed reduced G lignin, with increased S/G ratios (Additional file 1: Fig. S6, Table S4). These alterations are consistent with previous study that silencing of LAC17 affects the deposition of G lignin units [24]. Meanwhile, the recombinant PtoLAC14 protein showed higher oxidized efficiency to coniferyl alcohol (precursor of G lignin) in vitro. Taken together, these data implied that PtoLAC14 selectively promoted G unit deposition into lignin. The presence of expanded laccases in plant genomes advocates for divergence of functions in this gene family. Compared with grasses, woody plants have larger amounts of secondary cell wall and higher lignin content, indicating a complex mechanism may exist in woody biomass. A previous analysis has demonstrated that there are substantially larger amounts of 49 laccase genes in *Populus* compared to *Arabidopsis*, and all these *Populus* laccase genes had various expression patterns [31]. It is of interest to test whether these members have similar or reverse activities specific for monolignol polymerization in the future.

The content of lignin and the proportion of S/G ratio are crucial for the recalcitrance of lignocellulose in industrial use during enzymatic hydrolysis [15–18]. Plant laccases catalyze the last step of monolignols polymerization in lignin synthesis; it is possible to manipulate laccase gene expression for modifying lignin content and composition. In the present study, the *PtoLAC14*-KO plants, which contained lower lignin content and high S/G ratios, consistently exhibited improved biomass digestibility with little impact on plant growth (Fig. 6). Similar results have reported in previous studies, when knockdown of a laccase in *Populus* altered lignin content and increased sugar release [26], while inactivation of *LACCASE8* and *LACCASE5* genes in *Brachypodium distachyon* leads to 20–30% decrease in lignin content and high increase in saccharification yield without impacting plant integrity [33]. These results provided evidence that laccases are promising targets to enhance lignocellulose enzymatic hydrolysis. On the other hand, due to the functional redundancy of laccase members, the regulation of laccase by the upstream miRNA could also be efficient.

## Conclusion

The present study selected a novel dwarf (*df1*) mutant and identified it as a gain-of-function mutation of the *PtoLAC14*. With both genetic and biochemical evidence, we figured out the PtoLAC14 promotes lignification of cell wall, and selectively promoting the deposition of guaiacyl subunits. Importantly, *PtoLAC14*-KO plants showed significantly improved lignin S/G ratios, leading to remarkably enhanced biomass enzymatic saccharification. Hence, this study demonstrated that PtoLAC14 has specific functions in guaiacyl monomer polymerization, and provides a potent strategy for high cellulosic ethanol production by modifying lignin biosynthesis.

## Materials and methods

### Plant materials

Poplar (*Populus tomentosa* Carr.) plants were grown in a greenhouse under conditions of 16/8 h light/dark cycle with the 4500 lx supplementary light at 22*–*25*** °C*** and relative humidity ~ 60%. *Arabidopsis thaliana* plants were grown in a greenhouse under standard conditions at 22–24 °C with a 16-h day and 8-h dark cycle, with supplemental light 10,000 lx and 80% relative humidity. Measurement of plant growth was carried out on 10 plants for each WT and transgenic line.

### RNA extraction and PCR analysis

To determine the expression levels of *PtoLAC14*, RT-PCR and qRT-PCR analyses were performed. Total RNA from fresh tissues of poplar and *Arabidopsis* were extracted using RNA RNeasy Plant Mini Kit (Qiagen, Germany), and reverse-transcribed using an oligo-dT18 primer, RNase-free DNase (Takara, Dalian, China) and M-MLV Reverse Transcriptase (Promega Kit). Quantitative real-time polymerase chain reaction PCR (qPCR) was carried out on a Bio-rad MyCycler thermal cycler with the SYBR Premix ExTaq TM (Takara, Dalian, China) according to the manufacturer’s instruction. The poplar polyubiquitin gene (*PtoUBQ*) and *Arabidopsis AtGAPDH* were used as internal control. The relative quantification of the transcript levels was performed using the comparative Ct method. Each sample measurement was performed using at least two biological samples and each test of sample was conducted with three replicates. All primers used are listed in Additional file 1: Table S1.

### Vectors constructs and transformation

A FOX expression vector was produced according to the method described [29, 30] with slight modification. Briefly, total RNA from different tissues of poplar was isolated using RNeasy Plant Mini Kit (Qiagen, Germany). The poplar cDNA library constructed into plant expression vector pEarleyGate101 using a SuperSMART cDNA synthesis system in combination with the Gateway cloning technology.

The SALK T-DNA insertional line (*lac4*, GabiKat-720G02, AT2G38080) was obtained from the Salk Institute T-DNA insertion collection. A 2-kb fragment upstream of AtLAC4 start cordon was amplified from DNA with the primers ProAtLAC4-F/R. The full-length *PtoLAC14* cDNA was cloned from *P. tomentosa*, verified by sequencing, and inserted into the plant binary vector pCXSN under the control of *CaMV 35S* promoter or *AtLAC4* promoter.

The transgenic *Arabidopsis* plants were generated by introduction of the plant expression constructs (FOX vector and pAtLAC4::PtoLAC14) into an *Agrobacterium tumefaciens* strain GV3101 and transformation was done by floral dipping [39]. Two CRISPR/Cas9 target sites of *PtoLAC14* were assembled into binary pYLCRIPSR/Cas9 vector based on their GC abundance screened in the online tool ZiFiT TARGETER v.4.2 (http://zifit.partners.org/ZiFiT/Introduction.aspx) [36]. The constructs were introduced into *Agrobacterium tumefaciens* strain EHA105 and transferred into *P. tomentosa* by *Agrobacterium*-mediated transformation as described previously [40]. The transgenic lines were selected based on the hygromycin selection and PCR analysis. To identify CRISPR/Cas9-mediated mutation of *PtoLAC14* in transgenic poplar plants, the *PtoLAC14* genomic fragment was cloned into the pMD19-T vector (Takara) and at least 20 clones for each transgenic line were randomly selected for sequencing. All primers used are listed in Additional file 1: Table S1.

### Sequence alignment

All the database of the amino acid sequences was retrieved from the NCBI/GenBank/Blast sequence alignment. The GenBank accession numbers for genes used in this study are listed in Additional file 1: Table S2. Multiple alignments of full-length protein sequences were performed with ClustalW.

### Microscopic observation

Basal inflorescence stems (2–3 mm) of 6-week-old *Arabidopsis* plants and the sixth internode of the 5-month-old poplar stems were fixed in FAA buffer (formaldehyde:glacial acetic acid:50% ethanol, 1:1:18). After embedding in paraffin, the stems were cut into 5-μm cross sections using an ultra-thin semiautomatic microtome (FINESSE 325, Thermo). The cross sections were stained with 0.05% (w/v) toluidine blue for 5 min or 1% phloroglucinol–HCl (v/v) for 5 min. For Mäule staining, the stem sections were immersed in 1% (w/v) potassium permanganate solution for 5 min, then washed with water and acidified with 3% hydrochloric acid until partially decoloured, finally mounted in ammonia or 5% NaHCO_3_ and examined quickly [35]. The micrographs were taken under a Zeiss optical microscope (Zeiss, Oberkochen, Germany) and analyzed using IMAGEJ (https://imagej.nih.gov/ij/) for quantifying the morphological parameters of cells.

Scanning electron microscopy (SEM) was used to observe cell wall structures. Cross sections were obtained by dissecting transversely with razor blade by hand and the samples were attached using double-sided stick tapes. The samples were observed by SEM (PhenomtmPure FEI, USA) following the manual’s recommendations and images were captured digitally. Each sample was observed 10 times, and a representative image was used in this study.

### Plant cell wall fractionation and determination

For cell wall composition and biomass enzymatic hydrolysis analyses, around ~ 10 cm of the bottom part of the stems from 5 plants were harvested and the barks were peeled. The peeled stem samples were air-dried, the pith removed, the remaining tissues milled to a particle size of 20 mesh (0.85 mm), and the ground samples used for analyses. Plant cell wall fractionation and assay method were conducted as described previously [41, 42], all experiments were performed in the technical triplicates. After removal of soluble sugars, lipid, starch and pectin, the crude cell walls were suspended with the 4 M KOH containing NaBH_4_ (1.0 mg/mL), and washed three times with distilled water, the combined supernatants (KOH and distilled water) were used as KOH-extractable hemicelluloses fraction. The remaining pellets were dissolved in 72% H_2_SO_4_ (w/w) for 1 h at 25 ºC, and after centrifugation, the supernatants were collected to determine hexose as cellulose level. Total hemicelluloses were calculated by measuring hexose and pentose of the hemicellulose fraction and the pentose of the remaining cellulose pellets.

Total lignin content includes acid-insoluble and -soluble lignin was determined by two-step acid hydrolysis method as described [43]. The crude cell wall samples were hydrolyzed with 67% H_2_SO_4_ (v/v) at 25 °C for 90 min with a gentle shaking at 150 rpm, and subsequently diluted to 3.97% (w/w) with distilled water and heated at 115 °C for 60 min. The acid-soluble lignin was solubilized during the hydrolysis process, and was measured by UV spectroscopy at 205 nm. The remaining residues were placed in a muffle furnace at 575 ± 25 °C for 4 h for the acid-insoluble lignin assay. The lignin composition was isolated using thioacidolysis method and quantified by GC–MS [44].

### Chemical pretreatment and biomass enzymatic hydrolysis

Dried and milled poplar was used for analysis of sugar yield. Chemical pretreatment and sequential enzymatic hydrolysis were performed as described previously with minor modifications [45–47]. For H_2_SO_4_ pretreatment: the well-mixed biomass samples were treated with 6 mL 4% H_2_SO_4_ at 120 °C for 20 min, then shaken under 150 rpm at 50 °C for 2 h. For NaOH pretreatment: the well-mixed biomass samples were incubated with 6 mL 4% NaOH shaken at 50 °C for 2 h. After pretreatments, the pretreated residues were washed with distilled water for 3‒5 times until pH 7.0 for following enzymatic hydrolysis.

Enzymatic hydrolysis: the pretreated biomass residues were washed with mixed-cellulase reaction buffer (0.2 M acetic acid–sodium acetate, pH 4.8), then incubated with 6 mL (1.6 g/L) of mixed-cellulases (Imperial Jade Biotechnology Co., Ltd. Ningxia 750,002, China) co-supplied with 1% Tween-80. The sealed samples were shaken under 150 rpm for 60 h at 50 °C. After centrifugation at 3,000 g for 5 min, the supernatants were collected for pentoses and hexoses assay. All experiments were performed using ten representative plants in triplicate.

### Measurement of laccase activity

Leaf and stem samples were collected from three-leaf-stage seedlings of WT and transgenic lines. The samples were frozen in liquid nitrogen and quickly ground into a fine powder. Total proteins were extracted with the buffer (0.05 M borate buffer, pH 7.0, with 5 mM 2-mercaptoethanol and 2% (w/v) polyvinylpyrrolidone). The lysate was centrifuged at 13, 000 rpm for 20 min at 4 °C, and the supernatant contained crude enzyme extract. Total laccase activity was assayed as described [48] with ABTS (2,2-azino-bis3-ethylbenzothiazoline-6-sulfonic acid) as substrate by measuring the increase in absorbance at 420 nm. Protein concentration was determined by the Bradford method with bovine serum albumin (BSA) as standard, and one unit of enzyme activity is defined as 1 mg enzyme that oxidizes 1 nmol substrate per minute at 30 °C. Three independent biological replicates were performed for all samples.

### Expression and purification of recombinant PtoLAC14 protein

The coding region of PtoLAC14 gene was fused in frame with His in the pET-32a vector and expressed in E. coli (BL21). The cells were induced with 1 mM final concentration of isopropyl-1-thio-β-D-galactopyranoside (IPTG) for over-expression of recombinant proteins. After addition of IPTG, the cells were kept at 24 °C, 180 rpm for 16 h, and harvested by centrifugation at 7000 g, 4 °C for 10 min. The cell pellet was resuspended in 10 ml 50 mM sodium phosphate buffer, pH 7.0, with 300 mM NaCl. The resuspended cells were sonicated on ice for 10 min using 10 s off and 10 s on, 33% amplitude followed by centrifugation at 15,000 × g, 4 °C for 50 min, then the cell-free extract was separated from the cell pellet and filtered through 0.2 µm membrane. Purification of the recombinant proteins was carried out by Ni Sepharose 6 Fast Flow (Amersham Pharmacia Biotech) according to the manual’s recommendations. Recombinant protein samples were mixed with Roti®-Load 1 followed by incubation at 100 °C for 5 min, and analyzed by sodium dodecyl sulphate–polyacrylamide gel electrophoresis (SDS-PAGE) using 4.5% stacking gel and a 12% separating gel.

### Enzymatic oxidation of monolignols by recombinant PtoLAC14 protein

To evaluate the ability of recombinant PtoLAC14 protein to oxidize the monolignols, coniferyl alcohol (CAS No.: 458–35-5) and sinapyl alcohol (CAS No. 537–33-7) were used as substrates. The PtoLAC14 activity was assayed as described [49, 50] with minor modification. Each reaction contained 0.02 U enzyme, 0.5 mM of each substrates, and 50 mM NaAc–HAc buffer (pH 5.0, 30 °C). Absorbance of the reaction mixtures was monitored between 250 and 650 nm. The reaction mixture was scanned at 5, 10, 20, 30 and 60 min after reaction start, respectively, then terminated by adding NaN_3_. Water replacing the recombinant PtoLAC14 protein was used as the control in the reaction buffer with different mixtures and measured in the same time periods.

### Statistical analysis

Biological triplicate samples were collected for 10 plants of each transgenic line selection, and chemical analysis was performed in technical triplicates. The SPSS statistical software was used for data analysis. Statistical analysis was performed by Student’s *t*-tests (two-tail distribution and two samples with unequal variances) as *P < 0.05 and **P < 0.01.

## Supplementary Information

**Additional file 1: Table S1.** Gene-specific primers used for PCR amplification. **Table S2.** Information of related genes. Ptr: *P. trichocarpa*, At: *A. thaliana*, Sof: *Saccharum officinarum,* Bd: *Brachypodium distachyon.* **Table S3.** Cell wall composition (% biomass) of raw material. All data are given as means ± SD (*n* = 3, technical replicates). Statistical analyses were performed using Student’s *t* test as **P < 0.01 and *P < 0.05. **Table S4.** Lignin composition of raw material. **Fig. S1** Multiple alignment of *PtoLAC14* with other plant laccase proteins known to be involved in lignification. **Fig. S2** Expression of *PtoLAC14*. **Fig. S3** The expression profiling of *PtoLAC14* by qPCR analysis. YR, young root; MR, mature root; YL, young leaf; YL, ML, mature leaf; YS, young stem; MS, mature stem; X, xylem; B, bark. **Fig. S4** Generation of *PtoLAC14*-OE transgenic poplars. (a) Diagram of the *PtoLAC14*-OE vector. (b) The Hyg levels in the *PtoLAC14*-OE lines. (c) The expression levels of *PtoLAC14* in the *PtoLAC14*-OE lines. The poplar *ubiquitin* gene was used as an internal control. (d) Quantification of laccase activity with ABTS as the substrate. All data are given as means ± SD (*n* = 3, technical replicates). Statistical analyses were performed using Student’s *t* test as **P < 0.01. **Fig. S5** Collection of *PtoLAC14*-KO transgenic poplars. (a) Diagram of two CRISPR/Cas9 target sites of *PtoLAC14*. Target1 and Target2 indicate the positions of sgRNA-targeted sites. (b) DNA samples from independent transgenic poplar were analyzed for mutations by PCR assays. Primers are listed in Table S1. (c) Representative sequence at the target site. (d) Determination of mutations in transgenic poplar plants. (e) Determination of the mutations in the coding region of *PtoLAC14* generated by the CRISPR/Cas9 system. The text on the right summarizes mutation details in two independent CRISPR/Cas9-generated lines (L1 and L3). (f) Quantification of laccase activity with ABTS as the substrate. All data are given as means ± SD (*n* = 3, technical replicates). Statistical analyses were performed using Student’s *t* test as **P < 0.01. **Fig. S6** Lignin and monomer lignin in *PtoLAC14*-KO transgenic poplar lines. (a) Phloroglucinol–HCl staining of stem cross sections from WT and *PtoLAC14*-KO lines. Scale bars is 100 μm. (b) Mäule staining for lignin monomer composition of stem cross sections from WT and *PtoLAC14*-KO lines. Scale bars is 100 μm. (c) The monomer lignin S/G ratio in WT and *PtoLAC14*-KO lines. All data are given as means ± SD (*n* = 3, technical replicates). Student’s *t*-test was performed between the transgenic line and WT as **P < 0.01.

## Data Availability

All data generated or analyzed during this study are included in this published article and its additional file. Plant materials used in this study are available from corresponding author, Keming Luo (kemingl@swu.edu.cn).

## Abbreviations

LAC: Laccase
S: Syringyl
G: Guaiacyl
H: Hydroxyl-coumaryl
SEM: Scanning electron microscopy
Xy: Xylem
Xf: Xylem fiber cells
Xv: Xylem vessel cells
