# Supplementary material for: LACCASE14 is required for the deposition of guaiacyl lignin and affects cell wall digestibility in poplar
Source: Biotechnol Biofuels. 2020 Dec 3;13:197. doi: 10.1186/s13068-020-01843-4 (PMC7713150; doi:10.1186/s13068-020-01843-4)
Supplement: Supplementary file 1 — Additional file 1: Table S1. Gene-specific primers used for PCR amplification. Table S2. Information of related genes. Ptr: P. trichocarpa, At: A. thaliana, Sof: Saccharum officinarum, Bd: Brachypodium distachyon. Table S3. Cell wall composition (% biomass) of raw material. All data are given as means ± SD (n = 3, technical replicates). Statistical analyses were performed using Student’s t test as **P < 0.01 and *P < 0.05. Table S4. Lignin composition of raw material. Fig. S1 Multiple alignment of PtoLAC14 with other plant laccase proteins known to be involved in lignification. Fig. S2 Expression of PtoLAC14. Fig. S3 The expression profiling of PtoLAC14 by qPCR analysis. YR, young root; MR, mature root; YL, young leaf; YL, ML, mature leaf; YS, young stem; MS, mature stem; X, xylem; B, bark. Fig. S4 Generation of PtoLAC14-OE transgenic poplars. (a) Diagram of the PtoLAC14-OE vector. (b) The Hyg levels in the PtoLAC14-OE lines. (c) The expression levels of PtoLAC14 in the PtoLAC14-OE lines. The poplar ubiquitin gene was used as an internal control. (d) Quantification of laccase activity with ABTS as the substrate. All data are given as means ± SD (n = 3, technical replicates). Statistical analyses were performed using Student’s t test as **P < 0.01. Fig. S5 Collection of PtoLAC14-KO transgenic poplars. (a) Diagram of two CRISPR/Cas9 target sites of PtoLAC14. Target1 and Target2 indicate the positions of sgRNA-targeted sites. (b) DNA samples from independent transgenic poplar were analyzed for mutations by PCR assays. Primers are listed in Table S1. (c) Representative sequence at the target site. (d) Determination of mutations in transgenic poplar plants. (e) Determination of the mutations in the coding region of PtoLAC14 generated by the CRISPR/Cas9 system. The text on the right summarizes mutation details in two independent CRISPR/Cas9-generated lines (L1 and L3). (f) Quantification of laccase activity with ABTS as the substrate. All data are given as means ± SD (n = [file 13068_2020_1843_MOESM1_ESM.pdf]

Table S1. Gene-specific primers used for PCR amplification.

Name	Purpose	Forward primer (5'-3')	Reverse primer (5'-3')
FOX	FOX insertion gene cloning	TTTTACGACTCAATGACAAGA	CGATGGGGGTGTTCTGCTGGT
PtoLAC14	Gene cloning	CGGGATCCATGGAGTACGCTTGCTGGCTCC	CGGGATCCTCAACATTTTGGAAGGTCGCTT
	Cas9-Target1	GTCACCCTGCTTTGGTCCAGTGCA	AAACTGCACTGGACCAAAGCAGGG
	Cas9-Target2	GTCAGCGTTTAGGCAAGACAACCA	AAACTGGTTGTCTTGCCTAAACGC
	Cas9-Seq	GGCGATATGGAGTACGCTTG	ATTCAGCTAATACAACAACC
	Q-PCR	TGCCTCTTCCCTGCTTTGGTCC	GCCATTGACGGTAACAATCGGC
Hyg	PCR	ATCGGACGATTGCGTCGCATC	GTGTCACGTTGCAAGACCTG
PtoUBQ	Q-PCR	GTTGATTTTTGCTGGGAAGC	GATCTTGGCCTTCACGTTGT
ProAtLAC4	Promoter cloning	ACTGAGCTCACCATAGTTTTCATTGGAC	ATACCATGGCTCCCTCTCTATCTTTCTC
AtGAPDH	Q-PCR	GCAACATACGACGAAATCAAGAA	CGACACGAGAACTGTAACCCC

Table S2. Information of related genes.

No.	Genes	Accessions	Sequence identity to PtrLAC14	References
1	*PtrLAC2*	*Potri.001G184300.1*	61.24	Bryan *et al*., 2016
2	*PtrLAC3*	*Potri.001G206200.1*	67.26	Bryan *et al*., 2016
3	*PtrLAC14*	*Potri.006G096900.1*	100	Lu *et al*., 2013
4	*AtLac4*	*AT2G38080.1*	75.85	Berthet *et al*., 2011
5	*AtLac17*	*AT5G60020.1*	55.88	Berthet *et al*., 2011
6	*SofLAC*	*SCVPRZ3027A08.g*	57.54	Cesarino *et al*., 2013
7	*BdLAC5*	*Bradi1g66720.1*	56.99	Wang *et al*., 2015

Ptr: *P. trichocarpa*, At: *A. thaliana*, Sof: *Saccharum officinarum*,
Bd: *Brachypodium distachyon*.

Table S3. Cell wall composition (% dry matter) of raw material.

Samples	Cellulose	Hemicelluloses	Lignin
**WT1**	36.73±0.91	22.16±0.08	23.70±0.83
**OE-L1**	36.62±0.55	22.30±0.03	26.52±0.87
**OE-L2**	36.95±0.59	22.30±0.31	26.32±0.76
**OE-L5**	37.07±1.14	22.33±0.41	27.35±0.66
**WT2**	37.79±0.69	22.30±0.25	22.39±0.24
**KO-L1**	36.73±0.91	22.33±0.41	20.88±0.23
**KO-L3**	36.90±0.99	23.30±0.31	20.72±0.11

Table S4. Lignin composition of raw material.

Samples	Lignin monolignols (µmol/g biomass)			Lignin monolignols (% total)		
	G	S	H	G	S	H
**WT1**	143.42±3	203.45±8	1.44±0.1	41.18	58.41	0.41
**OE-L1**	160.05±4	195.28±5	1.64± 0.1	44.84	54.71	0.46
**OE-L2**	165.01±5	203.48±8	2.46±0.2	44.48	54.86	0.66
**OE-L5**	165.27±7	198.55±6	1.81±0.1	45.20	54.30	0.50
**WT2**	172.24±6	206.20±4	2.40± 0.2	45.23	54.14	0.63
**KO-L1**	154.64±5	207.97±5	2.41±0.2	42.36	56.97	0.66
**KO-L3**	155.20±5	210.82±6	2.71±0.3	42.09	57.17	0.74

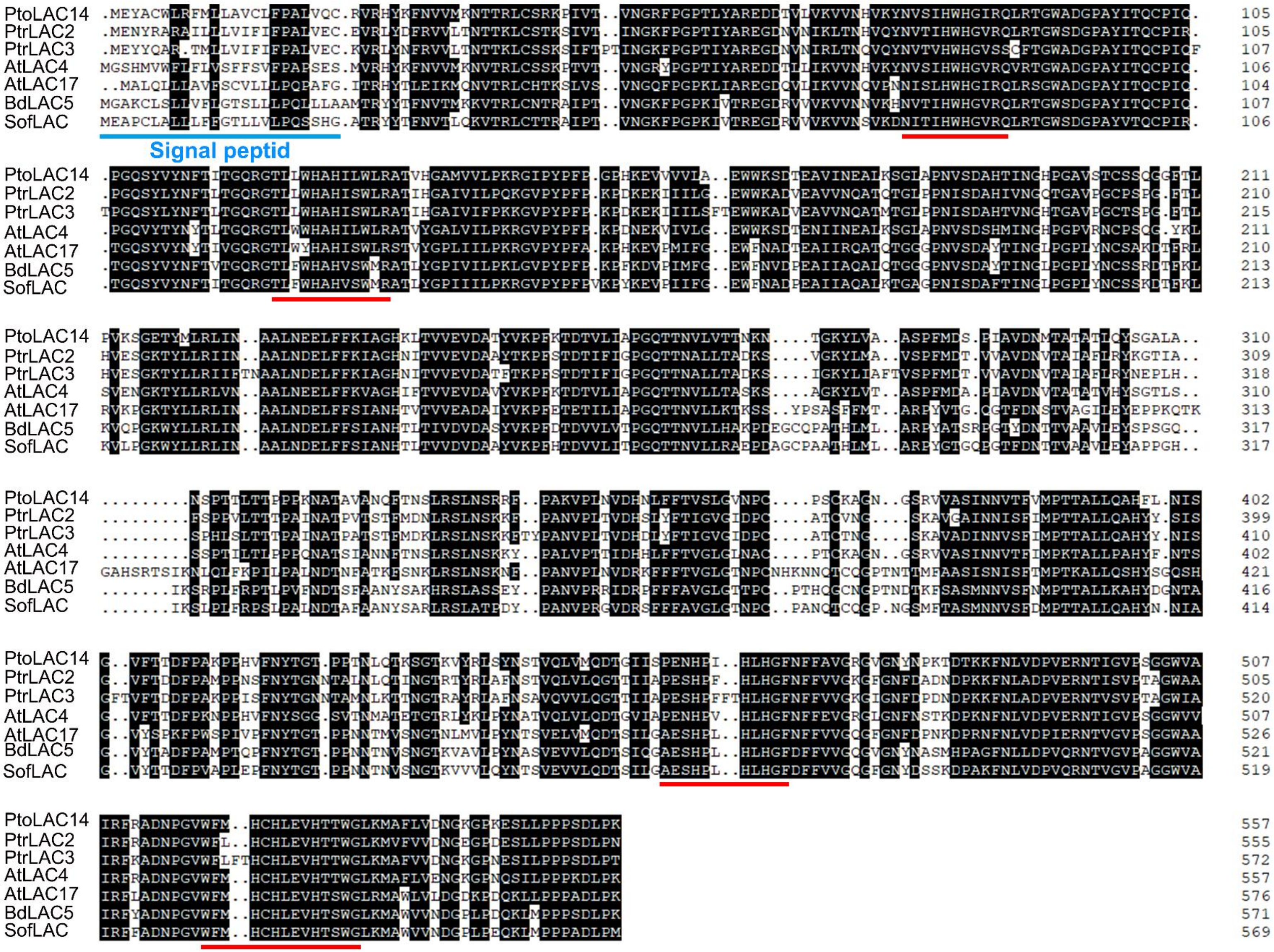

Fig. S1 Multiple alignment of *PtoLAC14* with other plant laccase proteins known to be involved in lignification. Ptr: *P. trichocarpa*, At: *A. thaliana*, Sof: *Saccharum officinarum*, Bd: *Brachypodium distachyon.*

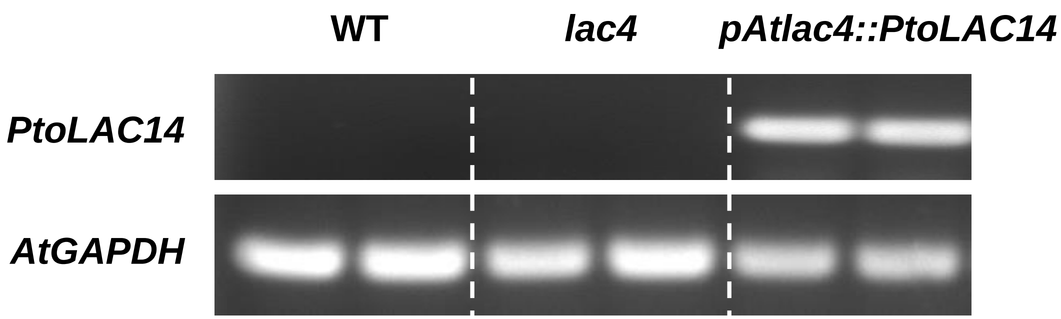

Fig. S2 Expression of *PtoLAC14*.

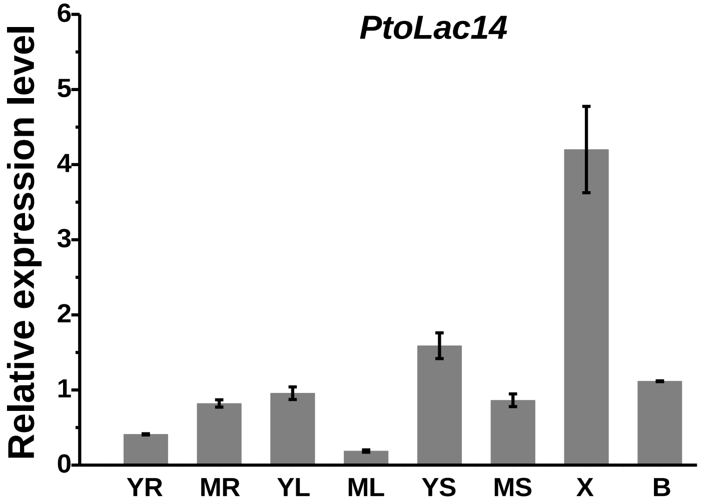

Fig. S3 The expression profiling of *PtoLAC14* by Q-PCR analysis.
YR, young root; MR, mature root; YL, young leaf; YL, ML, mature leaf; YS, young stem; MS, mature stem; X, xylem; B, bark.

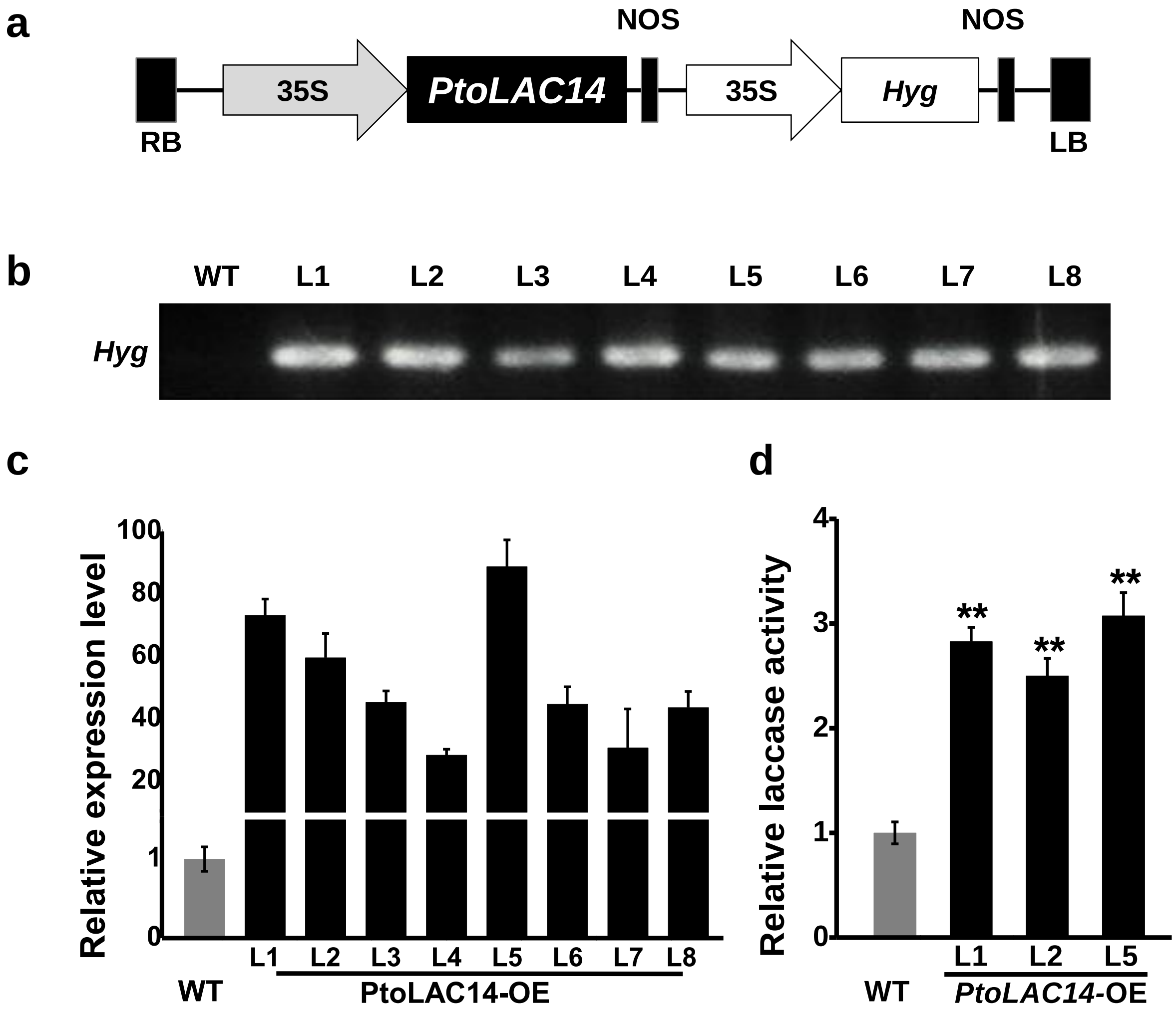

Fig. S4 Generation of PtoLAC14-OE transgenic poplars. (a) Diagram of the PtoLAC14-OE vector. (b) The Hyg levels in the PtoLAC14-OE lines. (c) The expression levels of PtoLAC14 in the PtoLAC14-OE lines. The poplar ubiquitin gene was used as an internal control. (d) Quantification of laccase activity with ABTS as the substrate. Data represent mean ± SD of three biological replicates. Statistical analyses were performed using Student's *t* test as $^{**}P < 0.01$.

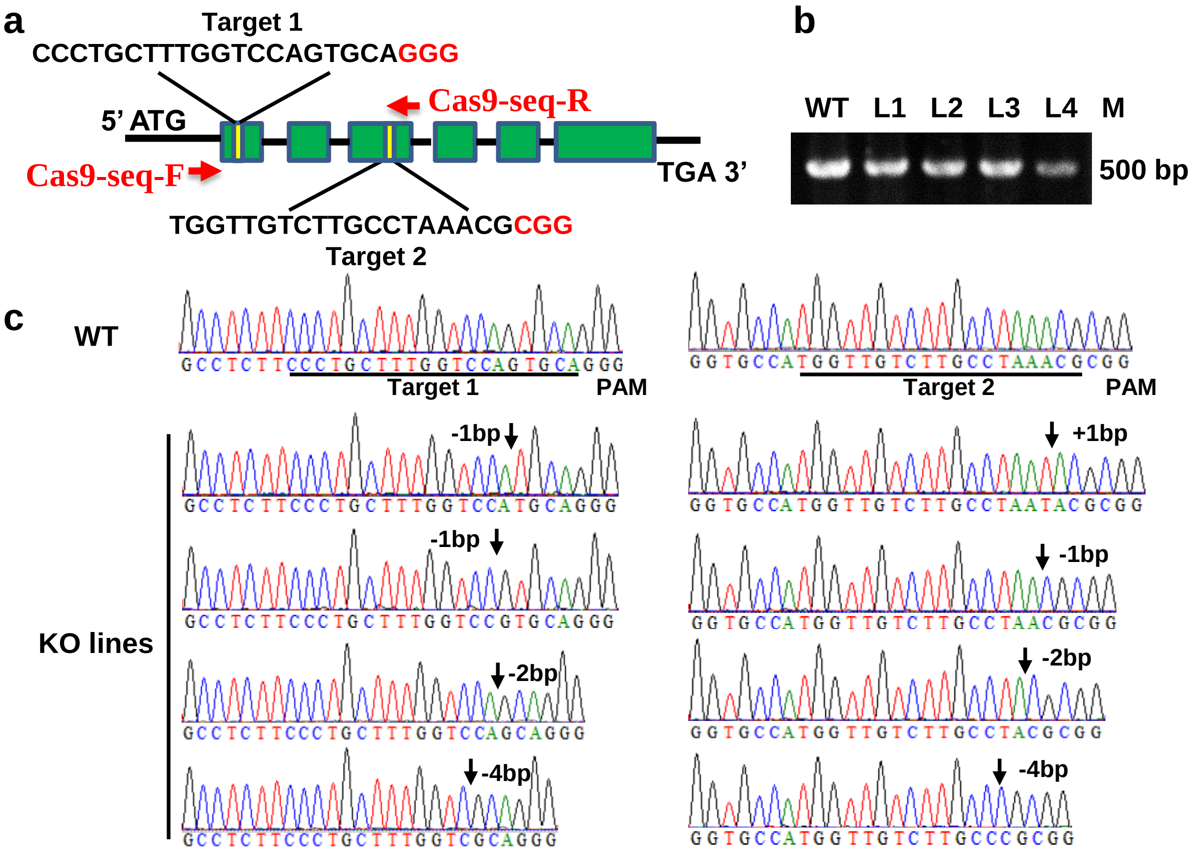

d

Target gene	No. of clones examined	No. of clones with mutations	Mutation rate (%)
PtoLAC14	50	42	84

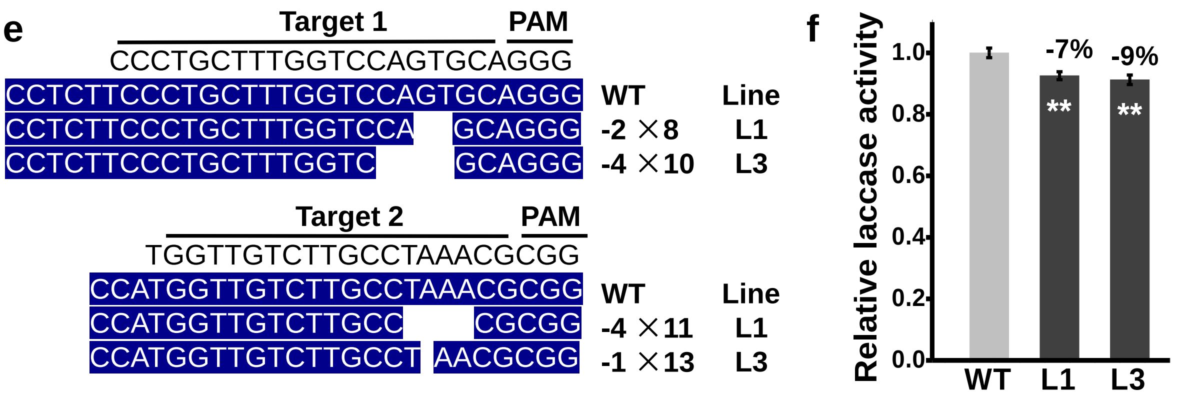

Fig. S5 Collection of *PtoLAC14*-KO transgenic poplars.

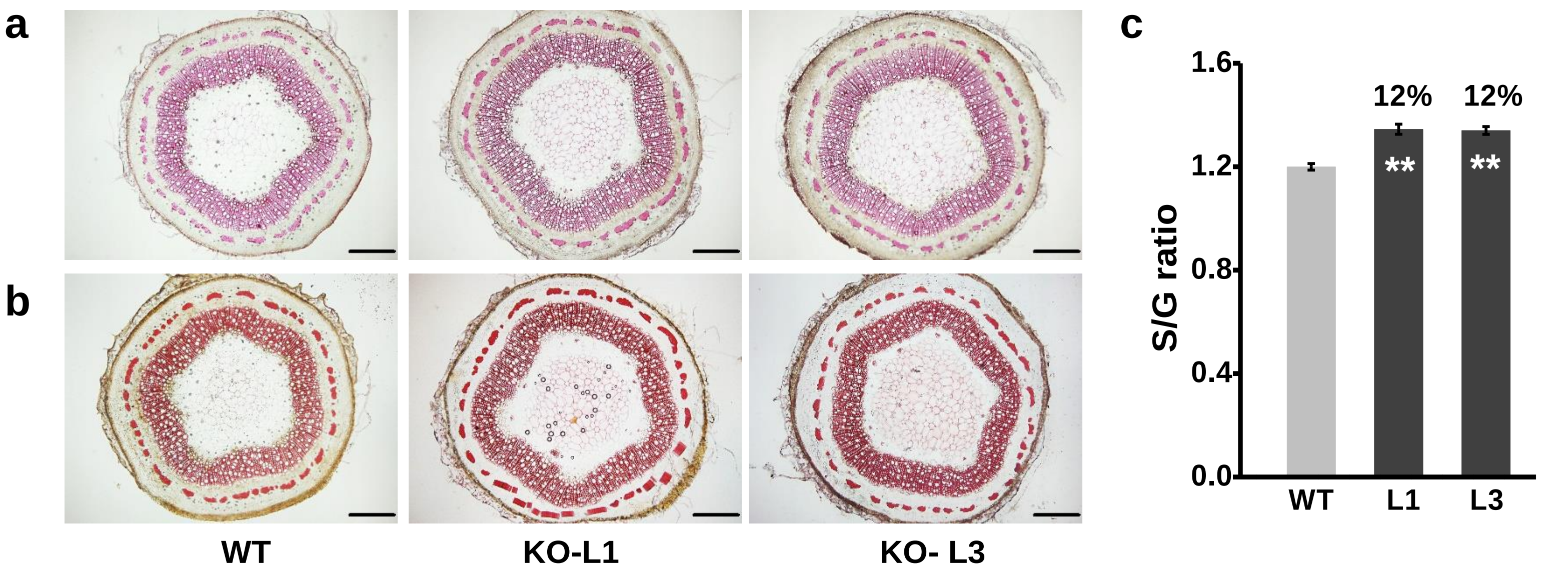

Fig. S6 Lignin and monomer lignin in *PtoLAC14*-KO transgenic poplar lines. (a) Phloroglucinol–HCl staining of stem cross sections from WT and *PtoLAC14*-KO lines. Scale bars as 100 μm. (b) Histochemical stained with Mäule reagent for lignin monomer composition of stem cross sections from WT and *PtoLAC14*-KO lines. Scale bars as 100 μm. (c) The monomer lignin S/G ratio in WT and *PtoLAC14*-KO lines. All data as means ± SD (n = 3). Student's *t*-test was performed between the transgenic line and WT as $^{**}P < 0.01$.
